# Effects of collagen matrix and bioreactor cultivation on cartilage regeneration of a full-thickness critical-size knee joint cartilage defects with subchondral bone damage in a rabbit model

**DOI:** 10.1371/journal.pone.0196779

**Published:** 2018-05-10

**Authors:** Kuo-Hwa Wang, Richard Wan, Li-Hsuan Chiu, Yu-Hui Tsai, Chia-Lang Fang, John F. Bowley, Kuan-Chou Chen, Hsin-Nung Shih, Wen-Fu Thomas Lai

**Affiliations:** 1 Graduate Institute of Clinical Medicine, College of Medicine, Taipei Medical University, Taipei, ROC; 2 Department of Obstetrics and Gynecology, Chung Kang branch, Cheng Ching Hospital, Taichung, Taiwan, ROC; 3 Department of Research, Taipei Medical University-Shaung-Ho Hospital, Taipei, Taiwan, ROC; 4 McLean Imaging Center, McLean Hospital, Harvard Medical School, Belmont, MA, United States of America; 5 Department of Pathology, School of Medicine, College of Medicine, Taipei Medical University, Taipei, Taiwan, ROC; 6 Restorative Dentistry and Biomaterials Sciences, Harvard School of Dental Medicine, Boston, MA, United States of America; 7 Department of Orthopedic Surgery, Chang Gung Memorial Hospital, Chang Gung University, Linkou Taoyuan, Taiwan, ROC; Kyoto Daigaku, JAPAN

## Abstract

Cartilage has limited self-repair ability. The purpose of this study was to investigate the effects of different species of collagen-engineered neocartilage for the treatment of critical-size defects in the articular joint in a rabbit model. Type II and I collagen obtained from rabbits and rats was mixed to form a scaffold. The type II/I collagen scaffold was then mixed with rabbit chondrocytes to biofabricate neocartilage constructs using a rotating cell culture system [three-dimensional (3D)-bioreactor]. The rabbit chondrocytes were mixed with rabbit collagen scaffold and rat collagen scaffold to form neoRBT (neo-rabbit cartilage) and neoRAT (neo-rat cartilage) constructs, respectively. The neocartilage matrix constructs were implanted into surgically created defects in rabbit knee chondyles, and histological examinations were performed after 2 and 3 months. Cartilage-like lacunae formation surrounding the chondrocytes was noted in the cell cultures. After 3 months, both the neoRBT and neoRAT groups showed cartilage-like repair tissue covering the 5-mm circular, 4-mm-deep defects that were created in the rabbit condyle and filled with neocartilage plugs. Reparative chondrocytes were aligned as apparent clusters in both the neoRAT and neoRBT groups. Both neoRBT and neoRAT cartilage repair demonstrated integration with healthy adjacent tissue; however, more integration was obtained using the neoRAT cartilage. Our data indicate that different species of type II/I collagen matrix and 3D bioreactor cultivation can facilitate cartilage engineering *in vitro* for the repair of critical-size defect.

## Introduction

Cartilage has little capacity for self-repair, although superficial defects in the subchondral plate heal with fibrocartilage to a limited degree [1]. The mechanical properties of subchondral fibrocartilage are inferior to those of normal articular hyaline cartilage, and injured joints are predisposed to continued arthritic degeneration [1, 2]. Current cartilage regeneration therapies include the placement of carbon plugs [3], periosteum [4], and periochondrium [5], in addition to autologous chondrocyte transplantation [3, 6–10] and subchondral drilling [11–13]. Success rates, however, vary widely and most methods have limited clinical use.

The structural, chemical, and mechanical properties of regenerated cartilage are not the same as those of normal cartilage [14], and even after multiple attempts, the regenerated tissue does not bond to adjacent tissue [15, 16]. New cartilage undergoes degenerative changes after 1 year, similar to healed tissue in untreated defects [16]. Thus, most repair methods fail to improve cartilage more than is achieved by natural repair of untreated osteochondral defects.

The need for improved treatment options for cartilage injuries has encouraged scientists to focus on *in vitro* implants from isolated chondrocytes [17–19]. Generally, chondrocyte proliferation *in vitro* with carrier matrices has included collagen gels [20–23], fibrin and hyaluronan [7, 24, 25], polyglycolic acid [26, 27], polyurethane microstructures [28, 29], and self-assembly in agarose gel [30].

Using a dynamic bioreactor system—a rotary cell culture system (RCCS)—is another approach to the regeneration of a cartilage defect and has been used to proliferate chondrocyte culture. This method successfully integrates cells [31]; however, it is unable to redifferentiate chondrogenesis [32].

Attempts have been made to enhance cartilage repair on the extracellular matrix or molecules. Either type I or type II collagen has been used as a scaffold to maintain the chondrogenic differentiation of chondrocytes [33, 34]. A study demonstrated that a type II/I collagen matrix plays a functional role in the regulation of chondrogenic differentiation using mesenchymal progenitor cells [35]. Furthermore, different collagen matrices may have varied effects on the maintenance of chondrogenic differentiation in chondrocytes.

The purpose of this study was (i) to evaluate the effects of different species of collagen matrices combined with RCCS on cartilage engineering [36], and (ii) to determine the efficacy of treating critical-size defects using neocartilage in a rabbit model.

## Materials and methods

### Type I collagen extraction and purification

Type I collagen was extracted and purified from the tendons of New Zealand white rabbits and the tails of rats, as previously described [19, 37, 38]. The tendons and tails were dissected, sliced, and washed several times using cold distilled water to remove plasma proteins and then extracted by constant stirring overnight at 4°C with 0.5 M NaCl in 50 mM Tris-HCl at pH 7.4. The supernatant was decanted, and the remainder was washed several times using cold distilled water to remove salts and subsequently incubated overnight at 4°C with 0.5 M HOAc (pH 2.5) to obtain the aqueous extract. A salt solution (0.9 M NaCl) was added to the extract, resulting in precipitation. The precipitate was collected using centrifugation at 13 000 rpm for 30 min and subsequently dissolved in 0.05 M HOAc to form a collagen-containing solution. Two other salt solutions (0.02 M Na_2_HPO_4_) were added to the collagen-containing solution over a 24–48-h period, causing precipitation. The precipitate was collected using centrifugation and dissolved in 50 mM HOAc to obtain another collagen-containing solution. This collagen-containing solution was dialyzed against 5 mM HOAc and finally lyophilized.

### Type II collagen extraction and purification

Type II collagen was prepared as previously described [35, 36]. Rabbit and rat cartilages were sliced and washed using 0.5 M NaCl with 20 mM EDTA in 0.05 M Tris buffer (pH 7.4). The glycoproteins were extracted using 4 M guanidine–HCl, and dissolved in 0.5 M acetic acid containing 1 mg/mL pepsin. The collagen was precipitated by adding 0.9 M NaCl, washed several times using 70% alcohol to completely remove the acid and salt, and resolved using 1 M NaCl in 0.05 M Tris buffer (pH 7.4). The supernatant was recovered, NaCl was added to achieve a concentration of 3.5 M, and the precipitate was then removed. Type II collagen was precipitated by adding NaCl to a concentration of 4.5 M and washed several times using 70% alcohol. The alcohol was removed, and the pellet was redissolved in 10 mM acetic acid to achieve a final concentration of 4 mg/mL. Both extraction and purification of type II/I was performed at the laminar flow hood and in the least contamination areas.

### Chondrocyte–collagen matrix construct and the RCCS

Chondrocytes were isolated from the articular cartilage of newborn New Zealand white rabbits according to previously described methods [36, 39, 40]. Tissue slices were incubated overnight in Hank’s balanced salt solution containing 1 mg/mL hyaluronidase and 1 mg/mL collagenase. After centrifugation, the cell pellet was resuspended in Dulbecco's modified Eagle's medium (DMEM) containing 10% fetal bovine serum, 50 μg/mL gentamicin sulfate, 100 units/mL penicillin G sodium, 100 μg/mL streptomycin sulfate, and 0.25 μg/mL amphotericin B. Next, 5 × 10^5^ cells were seeded per 10-cm Petri dish. Cells were cultured in a 5% CO_2_ incubator at 37°C. The medium was changed every 3–4 days, and cells were grown until subconfluent.

Both the type I and II collagen were gamma-ray-sterilized at an accumulated dose of 3 kGy and dissolved in 5 mM HOAc after sterilization. Both rabbit and rat type I and type II collagens were purified and partially digested using pepsin to remove telopeptides [37]. The type I- and type II-containing solutions were gently mixed and mildly heated to facilitate mixing if necessary. The ratio of type II to type I collagen was 1:4 [36].

An aliquot of 1.0 mL of 10^6^ chondrocytes was mixed with 1.0 mL of 4 mg/mL of collagen (type II/I) and placed in 24-well dishes until polymerization. Chondrocytes were respectively seeded in 10 rabbit and 10 rat collagen matrices. Chondrocyte–rabbit collagen matrix (neoRBT) and chondrocyte–rat collagen matrix (neoRAT) constructs were further incubated for 4–5 days at 37°C and in a 5% CO_2_ atmosphere, in DMEM supplemented with 20% fetal calf serum, Ham’s F-12K, 0.5 mM proline, 50 mg/L ascorbic acid, 0.2 mg/mL proteoglycans, 50 μg/mL gentamicin sulfate, 100 units/mL penicillin G sodium, 100 μg/mL streptomycin sulfate, and 0.25 μg/mL amphotericin B.

**Fig 1:**
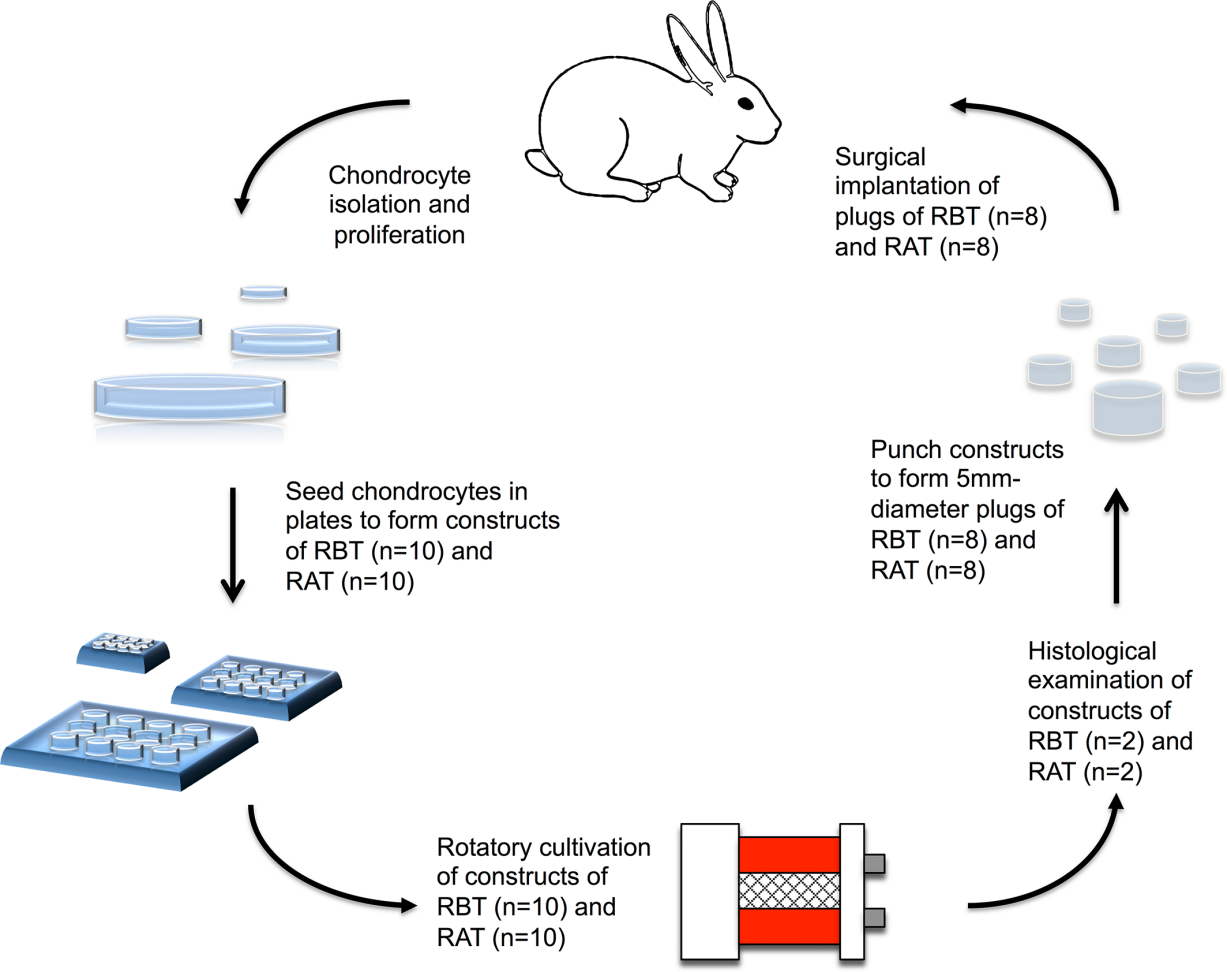
Experimental design. Chondrocytes were isolated from rabbit cartilage and proliferated in an RCCS. The cell-matrix constructs gradually formed neocartilage, and each was then embedded into a surgically created wound in the knee.

Twenty chondrocyte matrix constructs were first cultured in a flask for 4–5 days and then placed into a RCCS (RCCS-D; Synthecon, Texas) [36]. A gentle rotation of 10 rpm, with some degree of oscillation, was employed. The medium was changed every 2–4 days. The chondrocyte matrix formed neocartilage gradually over 2–4 weeks. The neocartilage was punched into a 5-mm-diameter cylindrically shaped implant for subsequent surgical implantation (Fig 1).

Two chondrocyte matrix constructs from each rabbit and rat were first removed from the vessel and flask to determine the degree of chondrogenic differentiation at 2 and 4 weeks. The constructs were grossly examined, fixed in 10% formalin, embedded in paraffin, and serially sectioned (Sacura Sledge microtome) at 5–10 μm. Tissue sections were stained with alcian blue and hematoxylin–eosin (HE). Histological examination of the chondrogenic differentiation of the constructs was performed under a microscope and recorded using photography. The remaining 16 chondrocyte matrix constructs were preserved for additional experiments in normal saline at 4°C.

### Rabbits

Thirty adult New Zealand male rabbits were housed in well-ventilated cages and fed a regular diet (Purina Rabbit Chow, Purina Mills, St. Louis, MO). The average age of the rabbits was 3 months, and their average weight was 2.0 kg. All animal experiments, monitoring, diet, and environmental control followed the standard operating procedures for laboratory animal breeding in accordance with Taiwanese legislation on the protection of experimental animals and the National Institutes of Health Guidelines for the Care and Use of Laboratory Animals (8^th^ Edition, 2011, www.nap.edu). All animal experiments were approved by the Animal Care Committee of Taipei Medical University.

### Preliminary study of full-thickness critical-size cartilage defects

Twelve male New Zealand white rabbits were used for the determination of an appropriate size of critical-size cartilage defect. Rabbits were anesthetized using an intramuscular injection of a mixture of ketamine (100 mg/mL, 0.65 mL/kg of body weight) and xylazine (20 mg/mL, 0.30 mL/kg of body weight). The skin around the knee was shaved anteriorly and washed with iodine. A parapatellar medial approach was used to access the knee joint. The patella was dislocated and three different-sized defects were created on the femoral condyle using a drill to cut a hole through subchondral bone into the cancellous bone. A 3-mm circular, 3-mm-deep defect without implantation was created in four rabbits; a 4-mm circular, 3-mm-deep defect was created in four rabbits; and a 5-mm circular, 4-mm-deep defect was created in the remaining four rabbits. Half of the surgically created defects were on the right knees, and the other half were on the left knees. At 3 months, the rabbits were sacrificed. The knee joints were removed *en bloc*, fixed with formalin, decalcified, and stained with HE.

### Implantation of neoRAT and neoRBT cartilage in full-thickness critical-size cartilage defects

Thirty rabbits were included in the experiment: 28 for experimentation with critical-size defects and two as sham-operated controls. The patella was dislocated and a 5-mm circular full-thickness defect in the articular cartilage on the femoral condyle was made using a 5-mm drill to create a 4-mm-deep hole through subchondral bone and extended into cancellous bone in the bone marrow cavity; neocartilage was then implanted into this hole. Rabbit collagen matrix neocartilage (neoRBT cartilage) was then implanted into eight rabbits. Rat collagen matrix neocartilage (neoRAT cartilage) was implanted into eight rabbits. The other eight rabbits were the autograft and allograft groups. Both knees of these rabbits were subjected to surgery wherein a graft from the right knee was implanted into the left to form the autograft group, and a graft from the left knee of one rabbit was implanted into the right knee of a second rabbit to form the allograft group. The remaining four rabbits in the control group underwent surgery without implantation. The joint without surgery was designated the intact control group. Half of the defects were made on right joints, and the other half were made on left joints to avoid a confounder effect.

To evaluate the process of cartilage regeneration, rabbits implanted with neoRAT and neoRBT cartilage were sacrificed, with four rabbits examined at 2 months and 3 months respectively. To determine the effects of constructs in the cartilage regeneration, four autograft, four allograft, four surgery without implantation, and two sham-operated rabbits were also sacrificed at 3 months. The knee joints were removed *en bloc* and examined macroscopically. Each femoral condyle was evaluated grossly for shape, color, and contour and uniformity of the cartilage by two independent observers according to the criteria established [41] in S1 Table. The distal part of the femur was fixed with formalin, decalcified, and sagittal-sectioned perpendicular to the defect. Sections were obtained from the center of the defects. The specimens were stained using HE and examined microscopically.

### Histological examination

Knee joint tissues with implants were coronally excised *en bloc*. Specimens were fixed in formalin, decalcified, embedded in paraffin, and serially sectioned (Sacura Sledge microtome) at 5–10 μm. Tissue sections were stained using HE. Tissue regeneration and/or fibrosis of the defective area were evaluated histologically. The following specific observations were made: (i) the host response was observed to evaluate the hosts’ foreign-body reaction; (ii) tissue regeneration and/or fibrosis, ossification, or calcification in the knee joint were determined; and (iii) the histological tissue response to the surgical trauma was identified.

### Statistical analyses

The data obtained were assessed for statistical differences using a paired *t* test to compare experimental characteristics to control.

## Results

### Determination of the full-thickness critical-size cartilage defect

**Fig 2:**
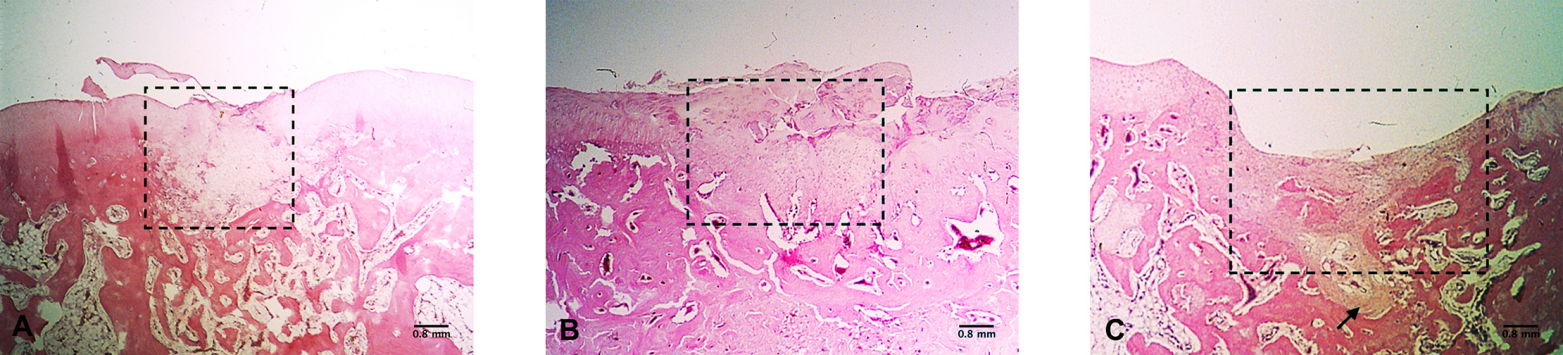
Various size bone defects at 3 months. (A) Self-repair was identified in the 3-mm circular, 3-mm-deep bone defects. Dotted box indicates the defect site. Note the surface disruption. (B) Fibrous cartilage repair with superficial fragmentation was apparent in the 4-mm circular, 3-mm-deep defects. Dotted box indicates the defect site. (C) Incomplete healing with fibrous tissue coverage was observed in the 5-mm circular, 4-mm-deep bone defects. Dotted box indicates the defect site. Note the fibrosis penetrating into the new bone formation region (indicated by arrow). All images: HE staining, magnification 20×.

The 3-mm circular, 3-mm-deep cartilage defects (Fig 2A) in the rabbit knee joint naturally self-repaired, whereas the 4-mm circular, 3-mm-deep cartilage defects (Fig 2B) only partially repaired themselves. However, the 5-mm circular, 4-mm-deep defects (Fig 2C) exhibited nonunion with severe fibrosis. Thus, a 5-mm circular, 4-mm-deep defect was used to be a standard for a critical-size cartilage defect.

### Neocartilage

**Fig 3:**
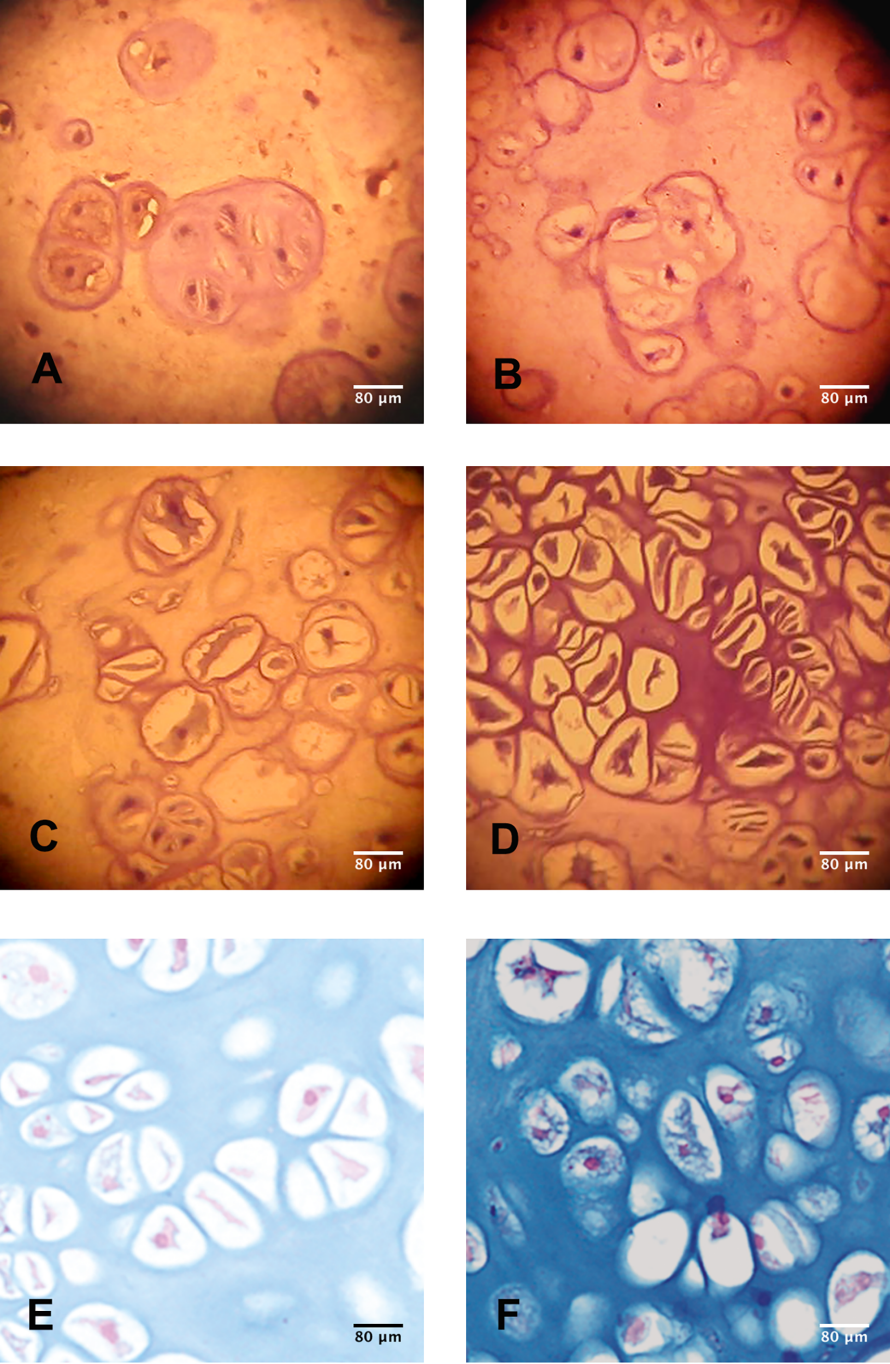
Rotating cell culture of chondrocytes for neocartilage constructs. Chondrocytes cultured in type II/I collagens from different species formed neocartilage constructs at 2 and 4 weeks (HE staining, magnification 200×). GAG expression of neoRBT and neoRAT cartilage at 4 weeks is displayed in the right-most column. Note the moderate GAG accumulation at 4 weeks in both the neoRBT and neoRAT cartilage. GAG accumulation around the lacuna of chondrocytes was observed in both neocartilage types.

Histology of the cell-matrix constructs revealed a cartilage-like appearance at 2 and 4 weeks. Chondrocyte–rabbit collagen matrix constructs exhibited mild chondrogenesis at 2 weeks and moderate chondrogenesis at 4 weeks (Fig 3A and 3C). Interestingly, chondrocyte–rat collagen matrix constructs in the RCCS showed mild to moderate chondrogenesis at 2 weeks and hypertrophic chondrogenesis at 4 weeks (Fig 3B and 3D). Moderate glycosaminoglycan (GAG) accumulation was discovered around the lacuna of chondrocytes at 4 weeks in neoRBT and neoRAT cartilage (Fig 3E and 3F).

### Macroscopic observation of the operated knees

**Fig 4:**
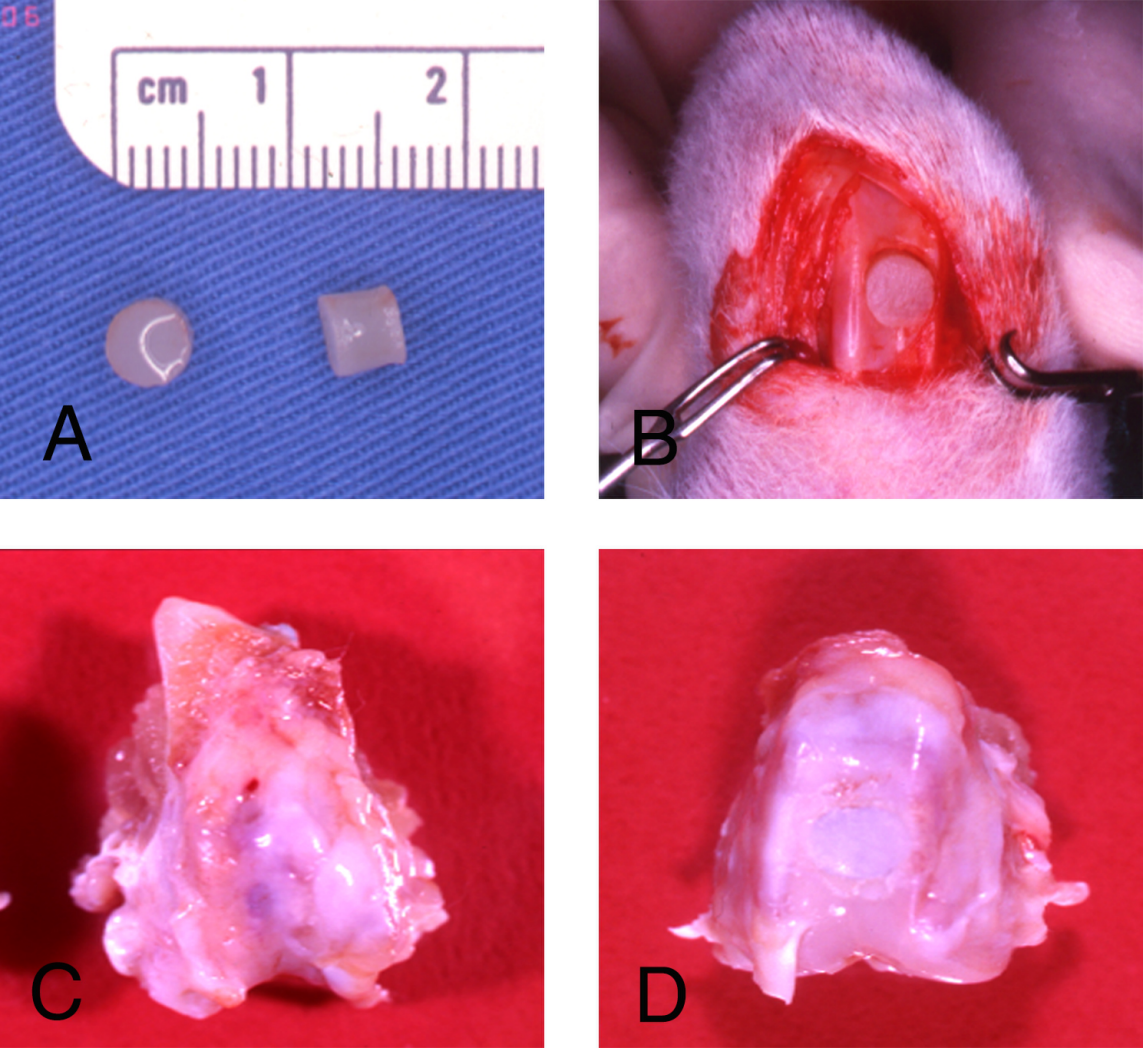
Neocartilage plug implantation. (A), (B) Neocartilage plugs of diameter 5 mm were implanted into the rabbit condyles. Condyles containing (C) neoRBT and (D) neoRAT cartilage at 3 months after surgery.

Chondrocyte–collagen matrices in the RCCS generally exhibited cartilage-like formation at 2–4 weeks (Fig 4A). The neocartilage appeared shrinking approximately one fourth at 2–4 weeks, and then was punched into a 5-mm circular, 4-mm-long plug for implantation (Fig 4A and 4B).

The groups of RAT, RBT, and autograft appeared normal articular cartilage like and semitransparently white with a yellowish cast and covered the articulating end of the femoral condyle at 3 months after surgery. The surgical defects without implantation at 3 months had irregular surfaces with fibrosis and were incompletely healed (Fig 4C).

No signs of osteoarthritis such as osteophytes, cartilage erosion, or synovial proliferation were observed in the operated knees of the neoRBT or neoRAT group. The defects were filled with opaque white tissue. At 3 months, both groups had cartilage-like tissue covering the surgical sites. The reparative tissue in the wound appeared semitransparent, and the margin was integrated with adjacent healthy tissue (Fig 4D).

The macroscopic scoring showed a significant difference among groups of autogaft, RAT, RBT, and the group of allograft, and the group of without implantation (S1 Table and S1 Fig).

### Histological evaluation of neoRAT and neoRBT cartilage implants

**Fig 5:**
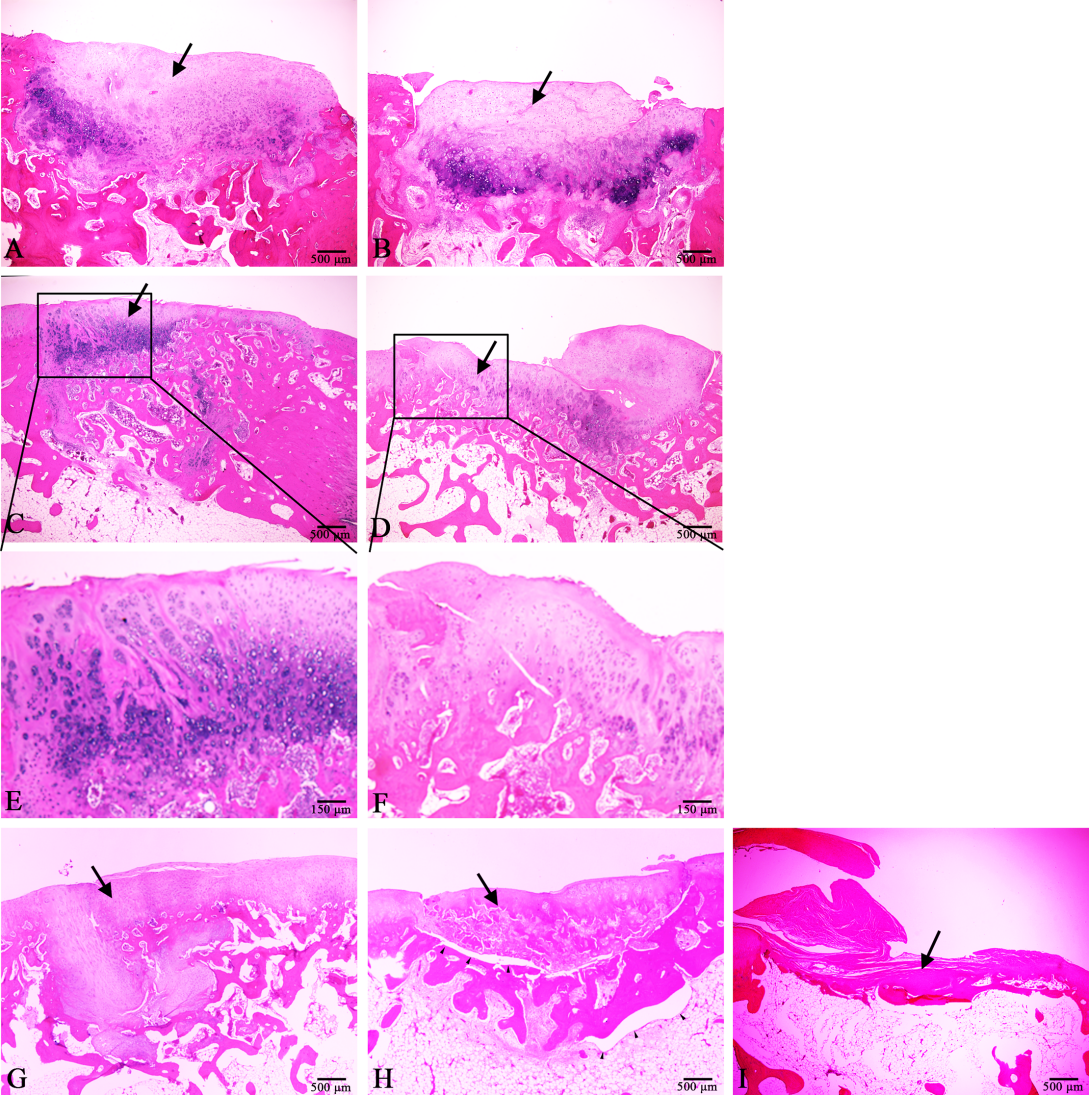
Cartilage regeneration using different neocartilage constructs. Black arrow in each graph indicates the defect site with or without implantation. In 2 months, less fragmentation and more integration with adjacent tissue were identified in neoRAT cartilage sites (A) compared with neoRBT cartilage sites (B). In 3 months, both (C) neoRAT and (D) neoRBT cartilage groups exhibited hyaline-like cartilage characterized by well-defined chondrocytes. Magnification of the labeled area in (C) and (D) revealed abundant aligned clusters in the (E) neoRAT and (F) neoRBT cartilage. The autograft group showed abundant hyaline-like cartilage penetration at 3 months. Well-defined wound healing was observed in the autograft group (G), whereas a gap was found between the base of the defect and the graft at 3 months in the allograft group (H). The allograft did not integrate with the adjacent normal tissue. In the surgery without implantation control group, the defect was filled with severe fibrosis, no cartilage was found in the defect area, the subchondral bone in the wound was denuded, no union had occurred, and pannus formation was noted in the defect area (I). All images: HE staining, magnification 20×.

In the neoRBT and neoRAT cartilage groups, the defects were predominantly filled with chondroblasts at 2 months. The cellular morphology varied from round to polygonal-like chondrocytes, and chondroblasts had penetrated into the subchondral layer. Mild inflammation was noted in the bottom of the defect area in both implantation sites. More fibrocartilage was discovered in neoRAT cartilage sites than in neoRBT cartilage sites. Mild fragmentation and less integration were identified in defects containing neoRBT cartilage, whereas integration with adjacent tissue was found in defects containing neoRAT cartilage (Fig 5A and 5B).

In the neoRAT and neoRBT groups, the defects were repaired by fibrous to hyaline-like cartilage at 3 months (Fig 5C and 5D). The neocartilage was thinner than the surrounding normal cartilage. Abundant, reactive, well-defined chondrocytes were found in the subchondral areas of both the neoRAT and neoRBT groups. The abundant chondrocytes had integrated with subchondral bone in the neoRAT group. Chondrocytes in the reparative cartilage were aligned in several apparent chondrocyte clusters in both groups. Remarkably aligned clusters were noted in both the neoRAT and neoRBT cartilage groups (Fig 5E and 5F). At the edges of the defects, both the neoRBT and neoRAT cartilage exhibited integration with adjacent healthy tissue; however, an irregular surface, moderate cleavage, and fragmentation of the cartilage were discovered in the neoRBT group.

### Histological examination of autograft, allograft implants and control groups

The autograft group, like the neoRAT cartilage group, exhibited abundant hyaline-like cartilage penetration at 3 months (Fig 5G). Both groups exhibited favorable wound healing and the wound sizes appeared to be small. The adjacent normal cartilage appeared to cross over to the defect areas. No inflammatory response was discovered in the autograft group, whereas mild inflammation was noted in the neoRAT and neoRBT cartilage groups.

The allografts did not integrate with adjacent normal tissue. A gap between the base of the defect and the graft was noted (Fig 5H). The allografts seemed viable, with no inflammatory reaction. The cartilage grafts exhibited no host-tissue reactions, but had not completely integrated with the defect tissue.

The defect was filled with severe fibrosis, and no cartilage was discovered in the defect area. The subchondral bone in the wound was denuded, and no union had occurred. A pannus formation was noted in the defect area (Fig 5I).

## Discussion

Tissue engineering has made great advances over the past decade and has shown great promise for the repair of cartilage defects. Numerous techniques for the production of cartilage that has the same qualities as natural cartilage have been studied, yet there remains obstacles to overcome [20–30, 35, 36, 42–48]. The biggest challenge for cartilage repair is the repair of a critical-size defect. In this study, we demonstrated how different components of a 3D substrate could be used to fabricate cartilage implants through embedding engineered chondrocytes. The substrate contains randomly rewound alpha-helical monomers of type I collagen and can further contain randomly rewound alpha-helical monomers of type II collagen. The cell-substrate construct is placed in culture medium. As chondrocytes proliferate and differentiate in the substrate, they secrete extracellular matrix proteins such as proteoglycans until a cartilage implant is produced. The collagen-based materials with the concentration of 4 mg/mL appeared shrinking approximately 1/4 of the total volume after 2–4 weeks. A punch of 5 mm diameter was performed after shrinking to obtain the implantation construct; therefore, the implantation constructs were completely fit the defect sites and may result in integration with adjacent cartilage tissue.

A previous study demonstrated that a critical-size defect—a 4-mm circular cartilage lesion—was repaired using collagen matrix (Enea *et al*., 2013). This agreed with the study of Messner, which discovered that a 3-mm circular, 3-mm-deep bone defect could self-repair in a rabbit model [49]. However, a 5-mm circular, 4-mm-deep defect only exhibited nonunion fibrous coverage at 3 months. Using this size of defect, reconstituted collagen matrix (types II/I) facilitated a regenerative effect during allograft implantation [50]. Specifically modified rat collagen matrix facilitated rabbit chondrocyte proliferation and preserved the phenotype in addition to the rabbit cartilage collagen matrix. Similar results were obtained in our previous study, which demonstrated regeneration of the temporomandibular joint disc using rat tail collagen matrices [38].

Chondrocytes, type I collagen, and type II collagen can be prepared from two or three animal sources [51, 52]. For instance, chondrocytes isolated from humans can be used in combination with bovine type I [33] and type II collagen. Chondrocytes embedded in the substrate, which are placed in a rotating and oscillating vessel, provide a permissive microenvironment for chondrogenesis (i.e., the formation of cartilage).

Kimura reported that chondrocytes embedded in collagen gel or matrix maintain the cartilage phenotype in long-term cultures [5]. In the present study, we evaluated cartilage formation in collagen matrices from different species and determined the efficacy of different 3D matrices to facilitate neocartilage implantation. Different matrix composition has been reported to contribute to a cartilaginous microenvironment [40, 53]. For example, collagen matrix contains a small amount of randomly rewound alpha-helical monomers of type I collagen due to degradation and also contains type II collagen synthesized by the embedded chondrocytes. The type I collagen in the 3D matrix strengthens the scaffold, whereas type II collagen facilitates chondrocyte proliferation, subsequently leading to neocartilage formation.

This study is the first to compare the collagen matrices of different species (rabbits and rats) with rabbit chondrocytes and to be implanted into the same species. When implanted into a cartilage defect, the neocartilage matrix adheres to the surface matrix of the adjacent tissue. Both the neoRAT cartilage and neoRBT appeared to have integrated with adjacent tissue, as occurred for the autograft. In contrast, the allograft exhibited a gap between the graft and the bottom of the defect. These results suggest that neocartilage constructs may provide more nutrients than an allograft and facilitate integration with adjacent normal cartilage. Collagen matrices of different species—rabbit chondrocytes with either rat or rabbit collagen—exhibited similar efficacy to the autograft of rabbit cartilage. These data demonstrated that the reconstituted collagen matrices (types I and II) can sustain the proliferation of chondrocytes and preserve their phenotype. This is in agreement with our previous study of a 3D *in vitro* model, which discovered that collagen type I/II matrices preserve the proliferation and differentiation of chondrocytes [23]. Interestingly, the neoRAT cartilage appeared to be more integrated with adjacent tissue than the neoRBT cartilage in this rabbit model. This may be caused by the different origin of the collagen matrices. It has been shown that the collagen fibril from rat-tail tendon is relatively straight and uniform in structure, whereas those from bovine tendon exhibit a heterogeneous pattern under AFM observation [54]. Moreover, rat-tail tendon collagen showed higher sensitivity to pepsin digestion (S2 Fig) compared to collagen from rabbit tendon [55]. These results imply that different origins may result in structural and biochemical differences of collagen matrices, resulting in variant tissue outcomes of integration at the implantation site.

The bioreactor cultivation of rabbit chondrocyte cells in a collagen matrix scaffold supported cell survival, differentiation, maturation, and cartilage matrix deposition. The results of the present study demonstrated that chondrocyte–collagen matrices produced using 3D RCCS generally appeared more hyaline cartilage-like tissue with lacunae formation surrounded the chondrocytes, which generated high-quality cartilage for implantation.

We presented a useful technique that employs matrices from different species combined with rotary cell cultivation to fabricate cartilage. The neoRAT and neoRBT cartilage were both discovered to significantly facilitate tissue regeneration in full-thickness critical-size cartilage defects in the rabbit model.

The variances in the capacity of cartilage regeneration using collagen from different species have not been fully evaluated in previous researches. Our novelty was to demonstrate and compare the regenerative effects of collagen matrices, fabricated from different species, which combine cell-matrix constructs underwent RCCS to treat critical-size cartilage defect. The different species of collagen matrix, rabbit chondrocyte-rat II/I collagen matrix and 3D bioreactor cultivation more significantly facilitates tissue regeneration and repairs the critical-size cartilage defect compared to those of same species of collagen matrix, rabbit chondrocyte-rabbit II/I collagen matrix in a rabbit model. The data yielded in this study may imply the clinical value of the tissue engineering approach. A longer evaluation timeframe will be further studied.

## Supporting information

S1 Table Macroscopic evaluation scoring of the operated knees at 3-month. The macroscopic scoring showed a significant difference among groups of autogaft, RAT, RBT, and the group of allograft, and the group of without implantation.
(DOCX)

S1 Fig Coverage, neocartilage, defect, and surface scoring of the operated knees at 3-month. The scoring on various parameters was plotted and compared between experimental groups. The mean±SD of respective experimental group was calculated and evaluated by t-test compared to surgery w/o implantation group. * P<0.05, ** p<0.005.
(DOCX)

S2 Fig Type I collagen from different species were analyzed on PAGE with or without pepsin digestion. Lane 1: marker; lane 2,4,6,8 are type I collagen from porcine, rabbit, rat and bovine. Lane 3,5,7,9 are these collagens after pepsin digestion at 37°C for 30 min. It shows that rat-origin and bovine-origin collagen has a relative high sensitivity to pepsin digestion compared to collagens from rabbit and porcine. M: marker; +p: with pepsin digestion; -p: without pepsin digestion.
(JPG)
