# Correction: Effects of collagen matrix and bioreactor cultivation on cartilage regeneration of a full-thickness critical-size knee joint cartilage defects with subchondral bone damage in a rabbit model

**DOI:** 10.1371/journal.pone.0199567

**Published:** 2018-06-18

**Authors:** Kuo-Hwa Wang, Richard Wan, Li-Hsuan Chiu, Yu-Hui Tsai, Chia-Lang Fang, John F. Bowley, Kuan-Chou Chen, Hsin-Nung Shih, Wen-Fu Thomas Lai

An affiliation for the seventh author is missing. Kuan-Chou Chen is also affiliated with: Department of Urology, Taipei Medical University-Shuang Ho Hospital, 235 New Taipei City, Taiwan, ROC.
