# Supplementary material for: Effects of collagen matrix and bioreactor cultivation on cartilage regeneration of a full-thickness critical-size knee joint cartilage defects with subchondral bone damage in a rabbit model
Source: PLoS One. 2018 May 10;13(5):e0196779. doi: 10.1371/journal.pone.0196779 (PMC5945026; doi:10.1371/journal.pone.0196779)
Supplement: S1 Table — The macroscopic scoring showed a significant difference among groups of autogaft, RAT, RBT, and the group of allograft, and the group of without implantation. (DOCX) [file pone.0196779.s001.docx]

**S1 Table: Macroscopic evaluation scoring of the operated knees at 3-month**

| Group | RAT | RBT | Autograft | Allograft | Surgery w/o  implantation |
| --- | --- | --- | --- | --- | --- |
| Macroscopic evaluation ^a^  Coverage | 4.00 ± 0.00 | 4.00 ± 0.00 | 4.00 ± 0.00 | 2.00 ± 0.82 | 0.50 ± 0.58 |
| Neocartilage color  Defect margins  Surface | 3.50 ± 0.58  4.00 ± 0.00  3.75 ± 0.58 | 3.25 ± 0.50  3.50 ± 0.58  3.50 ± 0.58 | 3.75 ± 0.50  4.00 ± 0.00  4.00 ± 0.00 | 1.75 ± 0.50  2.00 ± 0.82  1.75 ± 0.50 | 0.75 ± 0.50  0.50 ± 0.58  0.50 ± 0.58 |

^a^ Means ± SD; n=4

RAT=neoRat cartilage

RBT=neoRBT cartilage
