# Supplementary material for: Effects of collagen matrix and bioreactor cultivation on cartilage regeneration of a full-thickness critical-size knee joint cartilage defects with subchondral bone damage in a rabbit model
Source: PLoS One. 2018 May 10;13(5):e0196779. doi: 10.1371/journal.pone.0196779 (PMC5945026; doi:10.1371/journal.pone.0196779)
Supplement: S1 Fig — The scoring on various parameters was plotted and compared between experimental groups. The mean±SD of respective experimental group was calculated and evaluated by t-test compared to surgery w/o implantation group. * P<0.05, ** p<0.005. (DOCX) [file pone.0196779.s002.docx]

**S1 Figure: Coverage, neocartilage, defect, and surface scoring of the operated knees at 3-month**

The mean±SD of respective experimental group was calculated and evaluated by t-test compared to surgery w/o implantation group. * P<0.05, ** p<0.005.
