# Supplementary figures and images for: Effects of collagen matrix and bioreactor cultivation on cartilage regeneration of a full-thickness critical-size knee joint cartilage defects with subchondral bone damage in a rabbit model
Source: PLoS One. 2018 May 10;13(5):e0196779. doi: 10.1371/journal.pone.0196779 (PMC5945026; doi:10.1371/journal.pone.0196779)

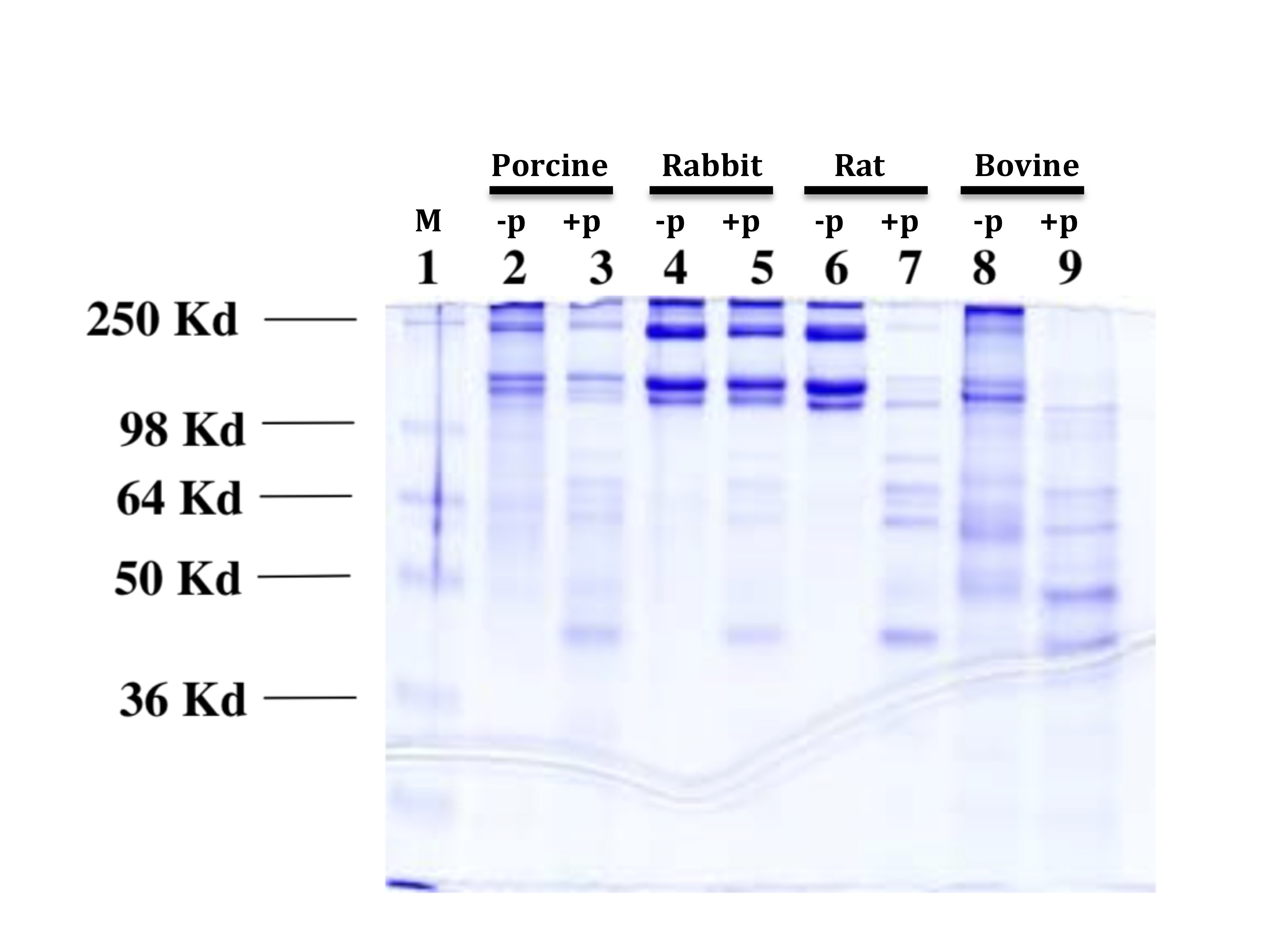

Supplement: S2 Fig — Lane 1: marker; lane 2,4,6,8 are type I collagen from porcine, rabbit, rat and bovine. Lane 3,5,7,9 are these collagens after pepsin digestion at 37°C for 30 min. It shows that rat-origin and bovine-origin collagen has a relative high sensitivity to pepsin digestion compared to collagens from rabbit and porcine. M: marker; +p: with pepsin digestion; -p: without pepsin digestion. (JPG) [file pone.0196779.s003.jpg]
